# A case-study evaluation of the “Copenhagen Music Program” for psilocybin-assisted therapy

**DOI:** 10.3389/fpsyg.2023.1156852

**Published:** 2023-06-16

**Authors:** Gina Ratkovic, Mike Sosteric, Tristan Sosteric

**Affiliations:** ^1^Lightning Path Institute, Sturgeon County, AB, Canada; ^2^Faculty of Humanities and Social Sciences, Athabasca University, Athabasca, AB, Canada; ^3^Faculty of Science, Athabasca University, Athabasca, AB, Canada

**Keywords:** case reports, guided imagery, music-informed psychedelic therapy, psychedelic therapy, psycholytic therapy

## Abstract

In a recent article, Messell and colleagues provide a curated list, the “Copenhagen Music Program for Psilocybin”. We test their music program with an experienced Indigenous therapist/psychonaut on a 3.5 gram psilocybin journey. Based on comments provided by the Indigenous therapist, we find the program contains musical choices that evoke specific colonial and religious contexts. We also find the program psychologically and emotionally coercive, meaning it is intended to contain the experience by forcing the individual on a specific experiential pathway. We conclude the program is not suitable for Indigenous travelers and suggest that curation of a wider variety of playlists, and music more in line with traditional shamanic practices, might be a better approach to psychedelic curation.

## Introduction

It has been long known that music is a critical factor influencing the psychological and emotional direction a psychedelic session takes (Gaston and Eagle, 1970; Bonny and Pahnke, 1972; Barrett et al., 2017, 2018; Kaelen et al., 2018). In the journal *Frontiers in Psychology*, in the research topic section entitled The *Psychotherapeutic Framing of Psychedelic Drug Administration*, authors Messell et al. (2022) provide a curated list of music (The Copenhagen Music Program for Psilocybin) that they feel accurately supports the underlying three-phase nature of the experience (Stenbæk et al., 2021). Their article offers procedural steps to creating such a list with the explicit aim being not to evaluate their own program but “to inspire others in their endeavors to create music programs for psychedelic interventions, while proposing that an informed music choice may support the therapeutic dynamics during acute effects of psilocybin” (Messell et al., 2022, para. 1).

Before approaching the task of selection, they set for themselves four criteria for music selection, that “(1) the music should reflect the intensity profile of a medium/high dose psilocybin, (2) the music should present cultural diversity of styles and genres, (3) vocal music pieces should avoid familiar languages, and (4) t*he music should avoid direct religious connotations*” (Messell et al., 2022, p. 2: italics added). As further guidance, they also note that “music which resonates with the patient’s experiences supports self-exploration,” that “liking the presented music” promotes safety and companionship, and that “openness to and liking the presented music. Correlate[s] with the intensity of acute psychoactive effects of psilocybin and with better antidepressant treatment outcome” (Messell et al., 2022, p. 2). After noting these important factors, they select a methodology for assessing the meaning in music (Hevner, 1936) and then individually and collectively set down to provide “meaningful sequences for the different sub-phases” of the psychedelic experience (Messell et al., 2022, p. 5).

## Evaluation

We evaluated the resulting “Copenhagen Program” with a case-study. An Indigenous female therapist, a member of our research and therapy team, and an experienced psychonaut with several decades of racial, sexual, and relationship trauma, ingested a 3.5 gram dose of dried Psilocybin mushrooms. The original goal of the experience, which was conducted in a specially designed, oft-used, sound-insulated specially designed space in our home, was personal and professional exploration of the effect of a high-dose psilocybin session. *There was no original intent to evaluate the Copenhagen program*, just to use it to enhance the experience, as the authors of the program (via their references to research indicating that music supports “self-exploration,” “safety and companionship,” intensity, and the ability to surrender and accept) implied that it might. Of course, as the authors of the Copenhagen Program suggested, their selection of music choices might not be appropriate in all cases. We were aware upfront that there may be issues, that the individual may not appreciate the music choices, that they may be triggering, but because our traveler was quite experienced with entheogenic substances, and because they had already accomplished a considerable amount of personal healing and reflection, we were not at all concerned over the potential for a disjunctive experience. We knew from experience that if there were issues they would be identified *in situ*, corrected, and the experience would continue. Our only hypothesis was that the music would provide a pleasant and fruitful variation on past experiences. Following ingestion, and at the first sign of psychological and emotional affect, we started the music program—about 30 min after ingestion.^1^

Unfortunately, the initial experience did not go well. The individual did not like the first pieces in the list, finding them “trite and inappropriate,” and generally resisting the music’s influence, as is sometimes the case (Kaelen et al., 2018). Thinking this distaste might reflect only the first few pieces, we quickly moved forward in the list, selecting the song “O magnum mysterium.” This, again, seemed a poor choice. The individual made critical comments on this piece, pointing out how it immediately placed her in the pews of a Catholic Church, a place where she, as an Indigenous victim of European colonization, had no desire to be. Still attempting to find some utility in the list, we randomly selected several additional pieces including “Dido and Aeneas” and the “Hymn of the Cherubim,” a piece with a Christian religious reference in the title, but again these pieces put the individual in the middle of a colonial religious and cultural framework. Continuing with our test, we then intentionally selected songs we felt would not evoke a known colonial frame, like “Om Namah Shivaya.” Once again the individual noted the inappropriate nature of a selection of Indian music, rejecting this choice as silly New Age appropriation. Her rejection was rooted in the awareness that new age spirituality is a commodified spirituality stripped of substance and turned exclusively toward the generation of private profit (Carrette and King, 2008). As our test progressed, the individual made additional comments concerning the jarring shifts of styles and continual and uncritical evocation of colonial culture. At the point where we found an Indigenous piece (“Calling the Other”), colonial undertones had been identified and trust in the program had been destroyed, leaving the Indigenous-themed selection completely out of place.

Unfortunately, we could not get through the list. Upon brief reflection during the experience, the individual suggested the soundtrack for the movie *Arrival*, a favorite movie of hers. It did not take long for our traveler to note, in contradistinction to the Copenhagen list, the gently evocative atmospheric nature of the soundtrack, so we immediately put that on loop for the duration of the journey. This *in situ* music choice provided a much better musical selection, immediately shifting the session, putting it back on an appropriate track, and allowing the individual to benefit from the rest of the experience.

## Context is key

As the authors of the curated list point out, music choice can amplify intercultural (and we might add intellectual/academic) power dynamics. This is exactly what happened during the experience. Several pieces in the curated list evoked and amplified colonial cultural paces, simultaneously invoking awareness of colonial institutions and colonial realities while putting our traveler smack dab in its ideological spaces. This disrupted the flow and undermined the journey. There was also the issue with jarring shifts of culture, genre, and style which also impacted the journey, repeatedly drawing the individual out of their experience.

Jarring shifts of style and insertion into colonial spaces were not the only problems with the list. In a subsequent debrief, the individual noted she was made immediately uncomfortable with the music which she felt was trying to force her to have a certain type of connection experience. This conclusion is somewhat surprising given that the Guided Imagery and Music (GIM) method inspired the author’s work (Bonny, 2002; Grocke, 2019). This inspirational method was developed by Bonny after she experienced a powerful mystical flow experience (Bakker, 2005) while playing violin at a social event. Her experience led to several insights, including a deeper appreciation for non-ordinary states of consciousness, the realization that she needed personal healing, *and* the realization that music, coupled with progressive Humanistic therapies, could facilitate this healing not only for herself but for others as well (Clark, 2002). She moved on to work with Pahnke and Grof where she consulted with researchers and helped choose music for LSD sessions (Clark, 2002). Bonny and Pahnke (1972) eventually published an article where they indicated music to be a crucial variable in the psychedelic process. Later, she developed the GIM program as a way to facilitate transformative experiences without the use of psychedelics (Grocke and Wigram, 2007), to “guide” an individual and get them “where they need to go” (Messell et al., 2022, p. 2). The explicitly stated intentions of GIM founders is not to direct the process but to leave it open to client direction, to trust the client’s unconsciousness, to support their “inner radar” (Grof, 2016) and to allow it to regulate itself. As Bonny notes in the “basic Premises of BMGIM,” all insight and healing comes from within (Clark, 2002). In this process the music selection is not intended to be coercive or rigid. The guide is expected to actively empathize with the traveler and actively modify the music program *in situ* as necessary to support the individual’s own direction (Clark, 2002).

The progressive intent of GIM founders and practitioners begs the question, why was this program experienced as coercive? The simple answer is that the coercion is baked in, though not intentionally so. The constructors of early psychedelic methodologies, the individuals involved in the development of Bonny method, were all of white Europe lineage. The theoreticians and mystics selected to ground the theory were also white. Bonny drew inspiration and nomenclature from Western psychologists like Maslow and Western mystics like Teresa of Avila and Evelyn Underhill (Bruscia and Grocke, 2002). If that is not sufficient to make the bias obvious, she was married to a theologically liberal preacher whose beliefs she resonated with (Clark, 2002). And it is not just the Western religious/mystical frame that we speak of here. Bonny and other psychedelic pioneers wrote at a time where European and capitalist perspectives were thoroughly hegemonic. Then, the mainstream accepted the good nature of the capitalist and his system just as one accepted the good nature of the priest and his Church. There was no questioning these perspectives within the mainstream. They were simply accepted as true. Therefore, approaches and methods constructed at that time were constructed within white-supremacist European rubrics which cast the ideal mystical and transformative experience within hegemonic Capitalist friendly, European-rooted frames. We anecdotally confirm this by browsing the appendices of the book *Guided Imagery and Music* where we find, not surprisingly, lists of Western music with consistent Western religious motifs (Bruscia and Grocke, 2002).

It would seem that, despite wishful claims to the contrary, music lists inspired by GIM work are shaped in ways that may not be immediately obvious to those working within these frames. There is no nefarious intent here. Your average liberal academic assumes that the sanitized Western-type mystical experiences, the standard Western scientific systems, and standard Western economic systems are superior to Indigenous systems (Sosteric, 2022). Why wouldn’t your average white, European researcher take an unquestioning look to the original GIM lists for inspiration? Why wouldn’t you use the music of upper-class Christian composers to guide the journey. Why wouldn’t you assume that the mystical-sounding Church organs of Western music practice were sufficient to elicit healing experiences? The whole thing is very Western, which is not to say that Western-type mystical experiences are not beneficial, just that unexamined ideological influences shape the development of music programs in Western ways, which in turn shapes the experience. And while your average Sunday-go-to-meeting white person might not experience a Church organ or modal music as coercive, that’s clearly not the case with those operating outside the colonial box. For them, the music program may be experienced as coercive. This may be viewed as an overstatement by some. As one reviewer suggested, it is not about coercing an experience, it is about providing a vehicle: “The music is a vehicle, and the listener’s consciousness is, ideally, the driver at the wheel.” It may be the case that the music *is* a vehicle, but one needs to keep in mind that not all vehicles are created equal. Some vehicles are designed for speed, some for off-road experiences, and some for destruction and war. It very much matters who built the vehicle and what its intended use are. A horse will get you places a car could never go.

## Containment

While it is important to understand that GIM programs are European Christian programs, this does not go far enough. It is also important to also understand that Christian mysticism and Western researchers influenced by patriarchal, capitalist, European hegemonic frames are functioning as purveyors of containment, reducing mystical experience from something much larger and more expansive to something much smaller and safer, for the *status quo*. These statements might sound odd to some, particularly as these experiences can expand one to the size of the universe, but it is not so odd when we realize that in addition to connecting one, in a contemplative, way with god, psychedelic experiences can also be highly emotive, sexual, and can lead to progressive insight, left-ward political shifting, ecological insight, and even revolution (Nour et al., 2017; Sosteric, 2018). Recall the revolutionary music of the sixties was rooted in psychedelic experience (Gilmore, 2016), or Dr. Timothy Leary telling people to “question authority” and “Tune in, turn on, and drop out” of the system. LSD and other psychedelics continue to fuel musical inspiration and development (Koroma, 2015; Music-News.Com, 2017). This aspect of psychedelic experience, the facilitation of critical political and ecological insight, action, and transformation, is at least as real and legitimate a goal for psychedelic therapy and, given the sorry state of the planet, at least as desirable as “personal transformation” or the development of gratefulness or “psychological flexibility” (Noorani, 2021). Yet all these aspects of mystical experience, particularly the political aspects, are not on the radar of modern psychedelic researchers who, instead of encouraging political or ecological enlightenment, simply ignore aspects of the field they find consciously or unconsciously undesirable.

Perhaps these comments are surprising, even objectionable, but they shouldn’t be. Colonized academics have been working to contain mystical experience to something pleasing to privileged white male European patriarchs ever since Stace dismissed aspects of the mystical experience that he, by his own admission, personally found distasteful, unpalatable, unbalanced, and irrational. In a glaring section of his book entitled “Discounting Raptures, Trances, and Hyperemotionalism” (Stace, 1960, pp. 51–55), Stace dismisses as inferior, hysterical, sexually frustrated stupidity any uncontained experience he finds too emotional, blissful, or rapturous.

But there can be no doubt that the abnormal bodily states which mystics call rapture or trance do sometimes occur. They are mentioned here as being of interest, but the point to be made is that they are accidental accompaniments of mystical consciousness, by no means universal or necessary. They occur among the more emotional and *hysterical mystics* and not among those of the more calm, serene, and intellectual types. They cannot therefore be regarded as belonging to the universal core of mystical experiences.

The same is to be said of the frequently asserted connection between sex and the mystical life; and of the sex metaphors which some mystics—especially in the Christian and Islamic traditions—lard their descriptions. It may well be true, as Leuba suggests, that a main part of the motives of St. Catherine of Genoa and Madame Guyon was the sex frustration which they underwent.” (Stace, 1960, pp. 52–53: italics added).

For Stace, the “correct” type of mystical experience is one that is “calm, serene, and unexcited,” one that brings not anger or revolutionary fervor, but “blessedness, bliss, joy, peace” (Stace, 1960, p. 55) and, lest we forget, forgiveness, because certainly you always want to forgive your oppressors. Anything else is discarded. Stace has been hugely influential and we see the impact of his containment comments quite clearly when we survey the available instrumentation (Hood, 1975; Hodge, 2003; Delaney, 2005), which consistently fails to operationalize the “distasteful” aspects of mystical experience (Abrams, 2016). We also see this when we contrast the carefully constructed and suitably sanitized clinical experience currently under development by mainstream actors (Abrams, 2016; Noorani, 2021) with a more politically charged effort, one that might facilitate an experience that connects one to the land, that explodes the consequences of colonialism, that brings political insight, that points in the direction of the obvious tragedy of consumer capitalism and its wholesale destruction of the planet, or that encourages social and political resistance. Such an approach would not rely on politically sterile classical music, nor would it eschew the use of meaningful lyrics and would perhaps use songs like Lennon’s “Working Class Hero” to bring awareness of toxic socialization (Sosteric and Ratkovic, 2016) or “The Trouble With Normal” by Bruce Cockburn. Such an experience would be more akin to the type of experiences common during the “uncontained” 1960s, experiences that lead to uncomfortable (for the accumulating class) political realizations and disruptive political actions which the “war on drugs” intentionally and effectively erased.

## Copenhagen containment

In the above context, and given the feedback provided by the Indigenous practitioner, we can clearly see the coercive and containing (Noorani, 2021) elements of the Copenhagen program. The Copenhagen music program is designed to facilitate medicalization (Noorani, 2020) while containing experiences not only within restrictive regulatory frameworks (Noorani, 2021) but also to the type of perennial, a-sexual, a-political, passive, peaceful and personal type of experience that is comfortable for the patriarchal, Neo-liberal therapeutic frames which seek, through the management of collective subjectivity (McHoul and Grace, 1993; Guttin, 2005), to suppress problematic ecological or political insights and angry revolutionary responses by turning the individual within were they can then blame their problems not on systemic issues like sexism, racism, violent socialization, or the exploitative Capitalist system, but on too-rigid thinking, lack of gratefulness, sexual frustration, insufficient “peace,” the absence of reason, or some other personal deficiency (Binkley, 2011). The coercive and containing nature of the program may not be immediately apparent to those working within Eurocentric frames, but it was obvious to our Indigenous psychonaut moments into the experience.

The authors of the Copenhagen article nod toward some of the issues identified above, suggesting their program might be criticized for being “too mechanistic” and for “not taking.the patient’s choice of music into account.” They also note that “When working with ethnic minorities or racial trauma.music choice can amplify intercultural power dynamics in the therapeutic relationship” (Messell et al., 2022, pp. 9–10). Anticipating these criticisms, they emphasize that “the effect of music must always be considered in relation to the listener’s history, preferences and cultural and social context of the listening experience” (Messell et al., 2022, pp. 9–10). All this is good advice, but it does not go far enough. In addition to considering the listener’s culture and history, white Europeans must also carefully consider their own history, which includes ongoing efforts to control the psychedelic/mystical experience. Proper attention here requires, at minimum, a certain level of sociological/political sophistication, a certain awareness of one’s own socio-cultural location (does one operate in the mainstream?), and a certain acknowledgment of one’s own cultural, often colonial, backgrounds and the biases and blind spots that these bring. Otherwise, we remain unconsciously complicit in ongoing efforts to control and contain human experience, a complicity that would be unfortunate given the remarkable potential of psychedelic therapy not only to heal, but to lead to social change and positive transformation.

It goes without saying, I suppose, that this critical self-reflection is important. Many specific demographics have been suppressed by the colonial systems that we often unwittingly represent, like women (Starhawk, 2011), or ethnic minorities who have been the subject of colonial and neo-colonial racism, or children who have been victimized by ecclesiastical predators. When you think about it, even privileged white males who have spent time in these institutions have been victimized. One of the authors of this paper is a *cis*-gender privileged white male, is still dealing with the trauma from childhood Catholic experiences and indoctrination. Even here it makes no sense to musically invoke Catholic frames.

## Discussion

Given the comments in this paper, rather than selecting for diversity in culture, style, and genre, if one is going to curate a list at all, it may be more appropriate to curate spiritually, culturally, and politically specific trip-lists with a more internally consistent selection of genre and style. Rather than aiming for the arid mystical experience preferred by a patriarch like Stace, perhaps we could aim, in addition to facilitating oneness and connection, for other targets, like facilitating political insight or connecting one ecologically with the land. Play lists like the short “Intentional Vibes,” “The Breath – Psychedelic Playlist 1,” Forest Sounds–Psychedelic Playlist 2,” or “Relaxing Piano–Psychedelic Playlist #3” could be used when individuals are seeking stereotypical spiritual experience, but other lists could be developed as well, like an ecological list, which might include songs like Michael Jackson’s “Earth Song,” or a political list, which might include Michael Jackson’s “They don’t really care about us” or the Who’s “Won’t get fooled again.” Curated lists like this would provide for a more ecologically valid set of options, more flexibility, and would put the power of choice more firmly in the hands of the participant rather than the psychedelic guides/researchers.

Speaking of putting the power into the hand of the person on the journey, even though curated lists of music are currently a thing in psychedelic therapy and research, all curated lists represent a containment of one type or another; all lists provide a specific vehicle with specific potential. As such, curated lists may not be the only way, or the best way, to approach the issue, particularly when using an approach like the GIM method which was developed within European colonial frames. Indeed, the coercion that underlies this approach may be in inherent conflict with the power of the psychedelic itself, with the individual’s own healing powers (which manifest, according to Grof, via an individual’s “inner radar” (Grof, 2016, p. 13) and with the more-than-human (MTH) beings which, according to Indigenous thinkers (Williams et al., 2022), help teach and heal. Indeed, the very idea of a curated list begs the question of cultural, political, and academic power. We need to be clear here. Curating a list of music enters the client immediately into an unequal power dynamic with the curators. This dynamic immediately privileges the curator and puts the power into their hands. Given this, perhaps we should be doing what Indigenous communities have been doing for many centuries with their shamanic practices, which is to use very simple music, drums, and atmospheric vocalizations that put the individual into a receptive space that persists throughout the journey. Setting such an auditory space would leave the power in the hands of the individual and would not force a traveler in a specific compass direction.

Note that the authors of the Copenhagen list suggest that there are times when distaste for the music expressed during a psychedelic session could be an issue of “transference” and “conflict,” and that the best way forward might be to “support and encourage the patient to stay with the music and engage the conflict.” This is certainly advisable in some cases; however, in the cultural and historical context of this study, this strategy would have been quite inappropriate. It would have reinforced centuries of colonial imposition (what do colonizers do but force their victims to engage with their cultural and political realities), undermined trust, “safety and companionship,” and completely destroyed the experience. In an individual with less experience, the forced imposition of the list might even have undermined trust in the drug itself, or the therapists, since a naive individual would be unlikely to connect the dots between the colonial music choices and the negative experience. The only appropriate way forward here would be to attend to the listener’s preferences and quickly select suitable musical alternatives.

We would also like to note that there is a need to shift burgeoning psychedelic methodology and research from a purely White-dominant medical/psychological framework to one that is critical, politically and sociologically sophisticated, *and* culturally inclusive (George et al., 2020). It is a relevant question whether authors would have curated a list of this nature had an Indigenous voice, or a sociologist, been included in the selection committee. It is reasonable to suggest that such inclusions (Williams et al., 2022) would have improved the committee’s curation.

Finally, this case study should not be seen as a specific criticism of the Copenhagen group, GIM, or even mainstream psychology, although all are culpable. The real culprit here is the Eurocentric, science-centric ideologies which cast as superior European ways of thinking and doing and which, as a consequence, facilitate and enable restriction, sanitation, and containment of everything, including therapy and research (Gibson and Beneduce, 2017). When Stace sanitized the mystical experience, when he presented what *he* was comfortable with as *the* exemplar, he thought we was doing the right. He thought that way because his ideology, his white, male, patriarchal, capitalist way of thinking convinced him that emotions were a sign of weakness and that the only valid experience was a politically and emotionally contained one. The take home here is not that some people got this “wrong,” but that that we all have to be more sensitive to our own biases and we all have to do a lot better job of unraveling the insidious influence of still powerful European hegemonic frames. To be clear, our target here is not Copenhagen or GIM specifically, but more generally the hegemonic European frames which dominate mainstream thinking and upon which GIM and Copenhagen rest. This is an important awareness. Would members of the Copenhagen team have selected the music they selected if they were aware of the conservative Christian roots of the GIM or the containing, mainstream nature of their activities? Would they have exercised more caution before releasing an untested program, would their warnings have been stronger, if they understood what their choices represented. We think not, which is why critical self and historical reflection is important, lest we contribute to the maintenance of a system that clearly has to go.

Finally, we would just like to highlight the fact that this paper represents a more general conversation between an Indigenous healer and the mainstream psychedelic research community. Comments in this paper come from an experienced Indigenous female therapist and psychonaut, one who immediately recognized the coercive “mainstream” flavor of the music and who rejected it as such. This rejection is not unreasonable. It fits well both with criticisms of modern psychedelic research which paint it as a containment effort designed to neutralize the political potentials of the experience *and* historical realities which did see uncontained usage lead to political insight and problematic (from the perspective of the accumulating classes) activation. Moving forward, it is up to individual researchers to decide whether they wish to submit to the containment agenda or pursue, dare we say, a more ecologically, psychologically, and spiritually valid approach to psychedelic experience and research.

## Data availability statement

The datasets presented in this article are not readily available because these were case-study notes and not of general interest. Requests to access the datasets should be directed to MS, mikes@athabascau.ca.

## Ethics statement

Ethical review and approval was not required for the study on human participants in accordance with the local legislation and institutional requirements. The patients/participants provided their written informed consent to participate in this study.

## Author contributions

TS, MS, and GR contributed to the conceptual analysis and content to the manuscript, wrote sections of the document, and were involved in editing and in the analysis of playlists. All authors contributed to the article and approved the submitted version.

## Footnotes

1 One reviewer of this article expressed concern that we were not more critical and cautious in our approach, or that, given the Indigenous background of the individual, that our hypothesis was that the experience would be positive and fruitful. The concerns may be coming from an assumption that our practitioner might have been put in danger by listening to some untested music. This would be a valid concern if this was a therapy client, or someone with minimal experience with psychedelics, but it wasn’t. This was an experienced psychonaut who was only *bothered* by the music choices. The music had no real power over this person, which is clear when you consider the chain of comments and criticism as we navigating the Copenhagen program. This person was not freaking out. There was no panic here, nor could there have been. There was no more danger here than there is when an able bodied child with a decade of walking experience walks to the store. That said, we would never had used this program with a naive traveler without a detailed intake and prior discussion of music choices, even in an experimental way.

## References

[B1] Abrams B. (2016). Development and evaluation of the Transpersonal Depth-Guided Imagery and Music (TD-GIM) inventory. *Nordic J. Music Therapy* 25 32–56. 10.1080/08098131.2015.1008557

[B2] Bakker A. B. (2005). Flow among music teachers and their students: the crossover of peak experiences. *J. Vocat. Behav.* 66 26–44. 10.1016/j.jvb.2003.11.001

[B3] Barrett F. Preller K. Kaelen M. (2018). Psychedelics and music: neuroscience and therapeutic implications. *Int. Rev. Psychiatry* 30 350–362. 10.1080/09540261.2018.1484342 30240282

[B4] Barrett F. S. Robbins H. Smooke D. Brown J. L. Griffiths R. R. (2017). Qualitative and quantitative features of music reported to support peak mystical experiences during psychedelic therapy sessions. *Front. Psychol.* 8:1238. 10.3389/fpsyg.2017.01238 28790944 PMC5524670

[B5] Binkley S. (2011). Happiness, positive psychology and the program of neoliberal governmentality. *Subjectivity* 4 371–394.

[B6] Bonny H. (2002). *Music and Consciousness: The Evolution of Guided Imagery and Music.* New Braunfels, TX: New Braunfels Barcelona Publishers.

[B7] Bonny H. L. Pahnke W. N. (1972). The use of music in psychedelic (LSD) psychotherapy. *J. Music Therapy* 9 64–87. 10.1093/jmt/9.2.64

[B8] Bruscia K. E. Grocke D. E. (eds) (2002). *Guided Imagery and Music: The Bonny Method and Beyond.* New Braunfels, TX: Barcelona Publishers.

[B9] Carrette J. King R. (2008). *Selling Spirituality: The Silent Takeover of Religion.* London: Routledge.

[B10] Clark M. (2002). “Evolution of the bonny method of guided imagery and music,” in *Guided Imagery and Music: The Bonny Method and Beyond*, eds Bruscia K. E. Grocke D. E. (New Braunfels, TX: Barcelona Publishers).

[B11] Delaney C. (2005). The spirituality scale: development and psychometric testing of a holistic instrument to assess the human spiritual dimension. *J. Holistic Nursing* 23 145–167. 10.1177/0898010105276180 15883463

[B12] Gaston E. T. Eagle C. T. Jr. (1970). The function of music in LSD therapy for alcoholic patients. *J. Music Therapy* 7 3–19.

[B13] George J. R. Michaels T. I. Sevelius J. Williams M. T. (2020). The psychedelic renaissance and the limitations of a White-dominant medical framework: a call for indigenous and ethnic minority inclusion. *J. Psychedelic Stud.* 4 4–15. 10.1556/2054.2019.015

[B14] Gibson N. C. Beneduce R. (2017). *Frantz Fanon, Psychiatry and Politics.* Lanham, MD: Rowman & Littlefield.

[B15] Gilmore M. (2016). *Beatles’ Acid Test: How LSD Opened the Door to “Revolver”.* Available online at: https://www.rollingstone.com/feature/beatles-acid-test-how-lsd-opened-the-door-to-revolver-251417/ (accessed February 12, 2023).

[B16] Grocke D. Wigram T. (2007). *Receptive Methods in Music Therapy: Techniques and Clinical Applications for Music Therapy Clinicians, Educators and Students.* London: Jessica Kingsley Publishers.

[B17] Grocke D. E. (2019). *Guided Imagery and Music: The Bonny Method and Beyond, Barcelona Publishers.* New Braunfels, TX: Barcelona Publishers.

[B18] Grof S. (2016). Psychology for the future: lessons from modern consciousness research. *Spiritual. Stud.* 2 3–36.

[B19] Guttin G. (2005). *Foucault: A Very Short Introduction.* Oxford: Oxford University Press.

[B20] Hevner K. (1936). Experimental studies of the elements of expression in music. *Am. J. Psychol.* 48 246–268. 10.2307/1415746

[B21] Hodge D. R. (2003). The intrinsic spirituality scale: a new six-item instrument for assessing the salience of spirituality as a motivational construct. *J. Soc. Service Res.* 30 41–61. 10.1300/J079v30n01_03

[B22] Hood R. W. (1975). The construction and preliminary validation of a measure of reported mystical experience. *J. Sci. Study Religion* 14:29.

[B23] Kaelen M. Giribaldi B. Raine J. Evans L. Timmerman C. Rodriguez N. (2018). The hidden therapist: evidence for a central role of music inpsychedelic therapy. *Psychopharmacology* 235 505–519. 10.1007/s00213-017-4820-5 29396616 PMC5893695

[B24] Koroma S. (2015). *A$AP Rocky Talks About the Influences Behind “LSD”.* Available online at: https://time.com/4100171/asap-rocky-lsd/ (accessed February 12, 2023).

[B25] McHoul A. Grace W. (1993). *A Foucault Primer: Discourse, Power and the Subject.* London: Routledge.

[B26] Messell C. Summer L. Beck Stenbaek D. S. (2022). Music programming for psilocybin-assisted therapy: guided imagery and music-informed perspectives. *Front. Psychol.* 17:873455. 10.3389/fpsyg.2022.873455 36467212 PMC9714440

[B27] Music-News.Com. (2017). *Wayne Coyne: “Miley Cyrus has done acid plenty”.* Available online at: https://www.music-news.com/news/UK/102703/Wayne-Coyne-Miley-Cyrus-has-done-acid-plenty (accessed February 12, 2023).

[B28] Noorani T. (2020). Making psychedelics into medicines: the politics and paradoxes of medicalization. *J. Psychedelic Stud.* 4 34–39. 10.1556/2054.2019.018

[B29] Noorani T. (2021). Containment matters: set and setting in contemporary psychedelic psychiatry. *Philosophy Psychiatry Psychol.* 28 201–216.

[B30] Nour M. M. Evans L. Carhart-Harris R. L. (2017). Psychedelics, personality and political perspectives. *J. Psychoactive Drugs* 49 182–191. 10.1080/02791072.2017.1312643 28443703

[B31] Sosteric M. (2018). Mystical experience and global revolution. *Athens J. Soc. Sci.* 5 235–255. 10.30958/ajss.5-3-1

[B32] Sosteric M. (2022). *What is Religion.* San Francisco, CA: Academia.edu.

[B33] Sosteric M. Ratkovic G. (2016). *Toxic Socialization.* San Francisco, CA: Academia.edu.

[B34] Stace W. T. (1960). *Mysticism and Philosophy.* New York, NY: Macmillan.

[B35] Starhawk. (2011). *Spiral Dance, The—20th Anniversary: A Rebirth of the Ancient Religion of the Goddess: 20th Anniversary Edition: Starhawk.* San Francisco, CA: Harper One.

[B36] Stenbæk D. S. Madsen M. K. Ozenne B. Kristiansen S. Burmester D. Erritzoe D. (2021). Brain serotonin 2A receptor binding predicts subjective temporal and mystical effects of psilocybin in healthy humans. *J. Psychopharmacol.* 35 459–468. 10.1177/0269881120959609 33501857

[B37] Williams K. Romero O. S. G. Braunstein M. Brant S. (2022). Indigenous philosophies and the “Psychedelic Renaissance.”. *Anthropol. Consciousness* 33 506–527. 10.1111/anoc.12161

